# Medium-to-long term sustainability of a health systems intervention to improve service readiness and quality of non-communicable disease (NCD) patient care and experience at primary care settings in Uganda

**DOI:** 10.1186/s12913-023-09983-7

**Published:** 2023-09-22

**Authors:** David Katende, Ivan Kasamba, Isaac Sekitoleko, Kevin Nakuya, Caleb Kusilika, Allan Buyinza, Michael Charles Mubiru, Gerald Mutungi, Moffat Nyirenda, Heiner Grosskurth, Kathy Baisley

**Affiliations:** 1 London School of Hygiene and Tropical Medicine, Bloomsbury, London, UK; 2 MRC/UVRI & LSHTM Uganda Research Unit, Entebbe, Uganda; 3 Ministry of Health Uganda, Kampala, Uganda

**Keywords:** Sustainability, Evaluation, Chronic diseases, NCDS, Health systems, Primary care, Sub-Saharan Africa, Uganda, Intervention trial, Long term, Medium term, Patient clubs, Adherence clubs

## Abstract

**Background:**

With the double burden of rising chronic non-communicable diseases (NCDs) and persistent infectious diseases facing sub-Saharan Africa, integrated health service delivery strategies among resource-poor countries are needed.

Our study explored the post-trial sustainability of a health system intervention to improve NCD care, introduced during a cluster randomised trial between 2013 and 2016 in Uganda, focusing on hypertension (HT) and type-2 diabetes mellitus (DM) services.

In 2020, 19 of 38 primary care health facilities (HFs) that constituted the trial’s original intervention arm until 2016 and 3 of 6 referral HFs that also received the intervention then, were evaluated on i) their facility performance (FPS) through health worker knowledge, and service availability and readiness (SAR), and ii) the quality-of-patient-care-and-experience (QoCE) received.

**Methods:**

Cross-sectional data from the original trial (2016) and our study (2020) were compared. FPS included a clinical knowledge test with 222 health workers: 131 (2016) and 91 (2020) and a five-element SAR assessment of all 22 HFs. QoCE assessment was performed among 420 patients: 88 (2016) and 332 (2020). Using a pair-matched approach, FPS and QoCE summary scores were compared. Linear and random effects Tobit regression models were also analysed.

**Results:**

The mean aggregate facility performance (FPS) in 2020 was lower than in 2016: 70.2 (95%CI = 66.0–74.5) vs. 74.8 (95%CI = 71.3–78.3) respectively, with no significant difference (*p* = 0.18). Mean scores declined in 4 of 5 SAR elements.

Overall FPS was negatively affected by rural or urban HF location relative to peri-urban HFs (*p* < 0.01). FPS was not independently predicted but patient club functionality showed weak association (*p* = 0.09).

QoCE declined slightly to 8.7 (95%CI = 8.4–91) in 2020 vs 9.5 (95%CI = 9.1–9.9) in 2016 (*p* = 0.02) while the proportion of patients receiving adequate quality care also declined slightly to 88.2% from 98.5% respectively, with no statistical difference (*p* = 0.20). Only the parent district weakly predicted QoCE (*p* = 0.05).

**Conclusions:**

Four years after the end of research-related support, overall facility performance had declined as expected because of the interrupted supplies and a decline in regular supervision. However, both service availability and readiness and quality of HT/DM care were surprisingly well preserved.

Sustainability of an NCD intervention in similar settings may remain achievable despite the funding instability following a trial’s end but organisational measures to prepare for the post-trial phase should be taken early on in the intervention process.

**Supplementary Information:**

The online version contains supplementary material available at 10.1186/s12913-023-09983-7.

## Introduction

Non-communicable diseases (NCDs) were predicted to account for over 70% of all disease burden in developing countries in 2020 as compared to just under 50% in 1990 [1]. The current burden of NCDs accounts for 71% of all global deaths (41 million people) each year [2]. Annually, 15 million NCD deaths occur between 30 and 69 years of age and over 85% of these "premature" deaths occur in low- and middle-income countries (LMICs) including Uganda [2]. In sub-Saharan Africa, between 1990 and 2017 the proportion of total disability adjusted life years (DALYs) attributable to NCDs increased from 19 to 30% of the total burden [2]. Therefore, health systems (HS) in these countries face the need to undergo a transition from services largely fashioned on managing communicable diseases like malaria, HIV/AIDS, or tuberculosis (TB) to services that must become able to address both these infections and the increasing burden of NCDs. Due to the urgency of care, the double burden on the already limited available resources means that curative services often get prioritised over preventative ones. Additionally, in LMICs, many NCDs including most cases of hypertension remain undetected due to the lack of active screening efforts [3, 4].

In Uganda, the prevalence of one or more modifiable NCD risk factors is over 94% indicating that NCDs may be largely preventable [5]. Despite the call to global action taken by the UN General Assembly resolution on NCDs control and prevention in 2011 [6], there is still limited domestic and global investment in addressing NCDs. Furthermore, the required response to NCDs among the poorest countries requires the introduction of integrated health service delivery strategies [7].

In 2016, we concluded a large cluster randomised trial to improve NCD care for hypertension (HT) and type-2 diabetes (DM) at primary care facilities in Uganda and Tanzania (the UK-MRC funded East African Chronic Disease Programme, EACDRP) [8]. The trial demonstrated significant improvements in NCD service readiness, with large differences between intervention and control facilities in the availability of functional basic equipment and healthcare worker knowledge. For example, in Uganda, the mean performance score in the intervention facilities was nearly double that in the control (74% vs 43%) and similarly 95% of these intervention facilities provided good quality NCD care according to national guidelines compared to only 8% in the control arm [8].

A comprehensive definition of sustainability of a health services intervention includes three components: (i) continued benefits to those who received the services when the interventions started and to new participants after the supporting funds were discontinued, (ii) continued implementation of intervention activities through a responsible organisation following discontinuation of financial support and (iii) community empowerment to improve their health by continuing intervention activities after its end [9]. Several efforts were made to ensure that NCD services were sustained after the EACDRP trial. These included close involvement of the ministerial and local governance structure in study activities, handover of important intervention resources (e.g., documents, equipment and up to 9 months’ buffer supply of NCD drugs at the end of the trial). The study also encouraged a patient-led initiative to form patient clubs which promoted peer support and contributions to a small-scale communal fund to assist patients with their treatment, by procuring drugs or supplies with a high stock out rate, e.g., bendrofluazide, metformin or glucose test strips over periods when the usual freely provided health facility (HF) supplies were interrupted.

The EACDRP created an excellent platform to assess medium-to-long term sustainability (which we defined as a period of 2 to 5 years after the end of research funding support) of a successful health system NCD intervention on the management of HT and DM within primary care settings in Uganda, the **MeLoHanD** study. We used this platform to re-evaluate health worker (HW) knowledge, and HT and DM service availability and readiness of HFs, and the quality of care experienced by patients at these HFs, at about 4 years after the research-related funding support had ended.

## Methods

### Study setting

Between 2013–2016, a cluster randomised trial of a health system intervention was conducted to improve NCD care in 38 primary care facilities and 6 referral facilities in Uganda (the EACDRP trial) [8]. The intervention package included the following components: training of health workers; provision of simple clinical management algorithms and patient registers in line with national guidelines; provision of essential NCD care drugs; active outpatient screening; promotion of NCD awareness and screening in the community outreaches, and regular support supervision visits to monitor the intervention conducted jointly by district and project staff.

**Fig. 1:**
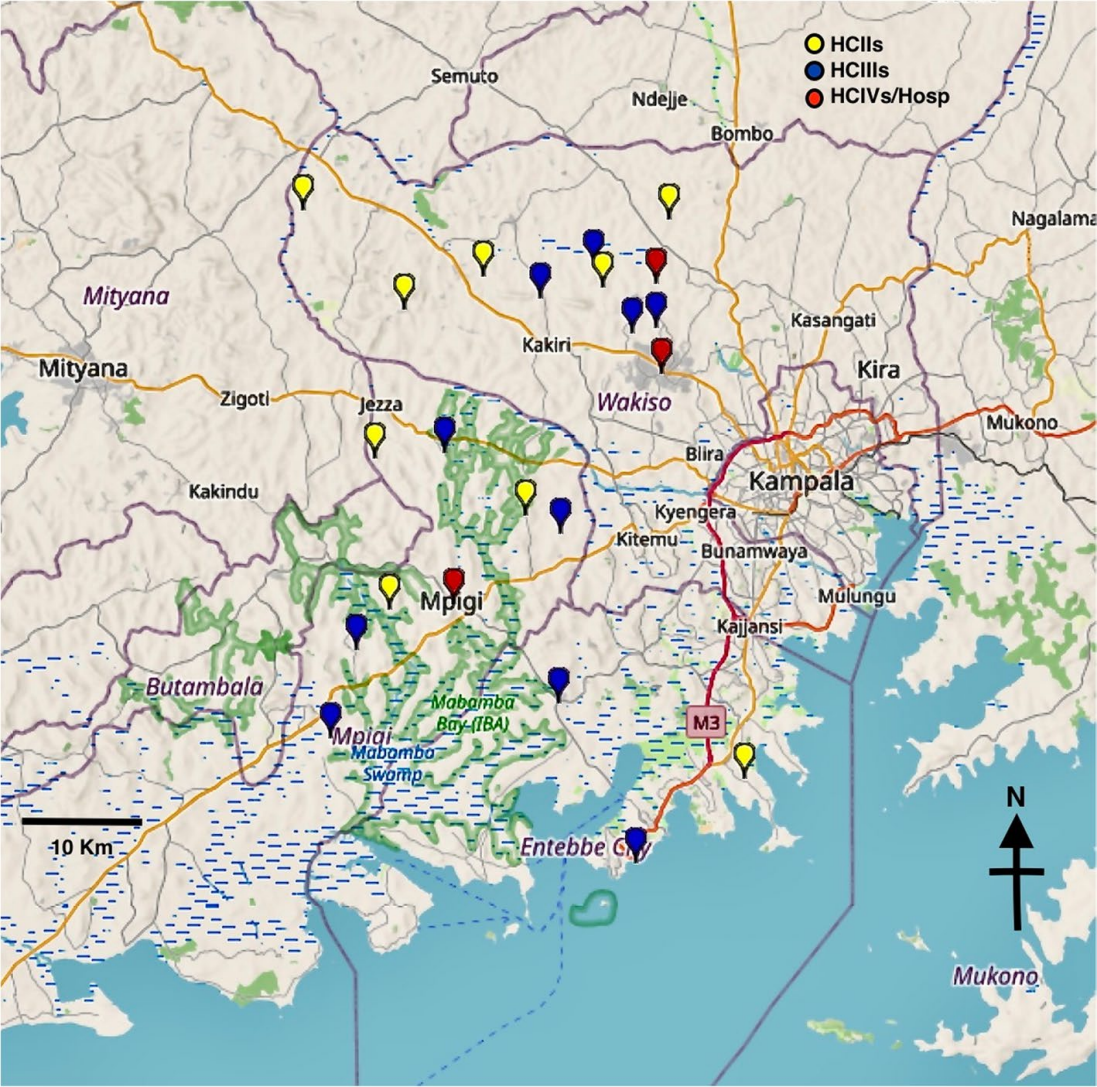
Map showing the distribution of participating health facilities across Mpigi and Wakiso Districts in Uganda (Developed using GPS visualizer.com with map data from OpenStreetMap.org, relief from ESRI/ArcGIS) *HC* Health centre levels II, III, IV, *Hosp* Hospital

At the end of the research support in 2016, an evaluation was conducted to assess HF service readiness and quality of patient care that the intervention project had achieved [8]. This involved a detailed inspection of each of the intervention and control facilities, a written test of HWs’ knowledge at each facility, and a survey of a random sample of 4 NCD patients from each facility. The surveys used standardised tools and questionnaires.

Fieldwork for the current MeLoHanD study was conducted between January and December 2020 in all 19 former intervention facilities and 3 referral facilities, in two central districts of Uganda: (a) Wakiso district, which forms a horseshoe shape around the capital city of Kampala (Fig. 1) with an urban, peri-urban, and rural population of 2.5 million [10]; (b) Mpigi district, which lies just southwest of Kampala along the shores of Lake Victoria (Fig. 1) and has a population of 250,000 [10]. The population is largely peri-urban and rural which is mainly engaged in subsistence farming, fishing, and artisanship.

### Description of health facility levels in Uganda

The primary health care system of Uganda is tiered along the politico-administrative organisation of the country (Table 1) and is overseen by the district health office, led by an experienced medical doctor, who co-ordinates resource distribution and staff deployment [11] to health centres (HC) II, III, IV and district hospitals (Fig. 1). Several districts form a region which is served by a hospital that can provide specialist care. HCIIs and HCIIIs which may include some private-not-for-profit health facilities are expected to diagnose and manage uncomplicated chronic disease cases including DM, HT, asthma, and HIV infection. HCIIs should also be able to diagnose DM, but usually refer DM patients to a HCIII or higher level facilities [12].

**Table 1:** Description of the levels of public health service delivery in Uganda

Health Facility Level^a^	Political or Administrative Level	Catchment Target Population	Main Function or Infrastructural Requirement	Facility head/Supervisor^b^ title and/or their educational background
Regional Hospital	Region or several districts	> 2 million	General and specialist services e.g., ophthalmology	Medical director (e.g., MD + MPH)
District Health Office	District	500,000 to 2 million	Resource distribution, staffing	DHO-er (e.g., MD + MPH)
District Hospital/HCIV	District or constituency	100,000 to 500,000	50–100 in-patient beds, general theatre,	Medical director (e.g., MD ± MPH)
HCIII	Sub-county	30,000	10–20 in-patient beds, maternity unit, a simple laboratory	Non-MD clinician or Mid-wife
HCII	Parish or several villages	5,000 to 10,000	1–2 day-care beds, first line emergency and out-patient care	Nurse
HCI	Village	1,000–5,000	Mobile outreach post	Nursing assistant or Health visitor

(Adapted from Readiness of Ugandan health services for the management of outpatients with chronic diseases, Katende et al., 2015)

^a^*HC* Health Centre, ^b^*MD* Medical Doctor, *MPH* Master’s course in public health

### Randomisation strategy applied during the original trial

Of the 38 Ugandan lower-level HFs (19 in each arm), 22 were randomised individually (as singletons) while the remaining 16 HCIIIs and nearby HCIIs (within a 10 km radius) were randomised jointly as 8 pairs, to minimise potential contamination owing to their proximity. In Uganda, hospitals and HCIVs are referral HFs for lower-level HFs in their catchment area. To ensure consistent care delivery to any patient referred to them from an intervention facility, all the 6 referral HFs (hospitals/HCIVs) in the project area therefore received the intervention without randomisation [13]. For each community in our study, the categories used for urban, peri-urban, or rural localisation followed the classification used by the Uganda’s 2010 national population census [14] and by the Uganda Demographic and Health Survey, 2011 [15].

### Study design

Our follow-up evaluation study (MeLoHanD) was a comparison of data from two cross-sectional surveys conducted at two-time points: the end of the EACDRP trial in 2016 and 4 years after the end of the trial, in 2020. We could thus evaluate the durability or sustainability of the service availability and readiness at former intervention HFs, as well as the health service-centred quality of care and patient experience (QoCE) over the 4 years without research-driven support.

The MeLoHanD study was conducted in 3 randomly selected higher-level facilities (HCIVs) of the 6 referral units, and all the 19 lower-level facilities (10 HCIIIs and 9 HCIIs) that constituted the original intervention arm of the EACDRP trial described in the section above (Fig. 1).

Of the 3 randomly selected HCIVs, 1 HCIV (peri-urban) was selected from Mpigi district while 2 HCIVs (1 peri-urban, 1 rural) were selected from Wakiso district. There were 7 lower-level facilities (4 HCIIIs, 3 HCIIs) from Mpigi and 12 lower-level facilities (6 HCIIIs, 6 HCIIs) from Wakiso district. Of these, only Wakiso district (Entebbe) had urban facilities (1 HCIII, 1 HCII) while the remaining 17 facilities (9 HCIIIs, 8HCIIs) were rural (Table 2).

**Table 2:** Distribution of health facilities by district and facility level

Facility attribute		District				Total
		**Mpigi**		**Wakiso**		
		HCIIIs	HCIIs	HCIIIs	HCIIs	
**Urban HFs**	*Singleton*	-	-	1	1	2
**Rural HFs**	*Singleton*	2	1	3	3	9
	*Paired*	2	2	2	2	8
**Referral facilities (HCIVs)**		1 *(Peri-urban)*		2 *(1 Peri-urban, 1 rural)*		3
**Total**		8		14		22

A study pilot to test study tools and prepare the study team for their field work was done in two health facilities (an urban HCIII and a peri-urban HCII) prior to this evaluation. Findings from these pilot HFs were not included in the analysis for the MeLoHanD study.

To evaluate *service availability and readiness*, each HF was inspected using a modified WHO Service Availability and Readiness Assessment (SARA) tool [16]. In addition, a clinical knowledge assessment was administered to all health workers present at the HF on the survey days. This is a validated knowledge test that has been used previously in East Africa [17, 18].

### Selection of participants for the MeLoHanD study

For the *quality of care and experience* (*QoCE)* assessment, patients were interviewed using a previously used tool as well as any available patient records to check consistency in responses. Twenty active patients who had been in care for at least 3 months at each HF were randomly selected from all HT and DM patients on the patient register. The study team made a pre-survey visit to each HF to confirm the survey days, prepare the HF staff, get the contacts of the identified individuals, and invite them on the survey days. We applied a restricted random selection approach to ensure adequate balance of men and women, and of HT and DM patients. Men and DM patients were found to be heavily under-represented in the study pilot.

All 20 selected patients were invited to visit the HF for participation in the survey. A transport refund was given to those that presented on the survey day. Efforts were made to contact those that did not present on the scheduled days to encourage their participation. We aimed to establish reasons for non-participation. Defaulters were not replaced. To ensure adequate statistical power for the study four times more patients were sampled in 2020 than in 2016. All assessment tools were identical to the versions used for the evaluation of the EACDRP intervention in 2016.

### Assessment of service availability and readiness (SAR)

The modified SARA tool assessed five aspects of service delivery elements (listed below). A HF could obtain up to 10 points for each element, so that a maximum of 50 points could be achieved.

i. Availability and functionality of essential equipment (e.g., blood pressure (BP) machine, weighing scale, stadiometer, glucometer/urine dipsticks, stethoscope, patient register book, HT/DM screening logbook, referral register book, health education record).
ii. Availability of drugs and other consumables (for the treatment of HT and DM and whether drugs were in line with guidelines and in sufficient stock).
iii. Quality of records on patients with NCDs (guidelines, essential demographic data, clinical observations, information on diagnosis, treatment, referral and follow up).
iv. Healthcare utilisation at facility by patients with NCDs (number of HT and DM patients, evidence for increase in utilisation over past year).
v. Prevention of NCDs and their complications (evidence for and number of health education sessions given, evidence for active screening for HT or DM among those that presented with other conditions e.g., back pain evidence of outreach activities).

Details of the adapted SARA tool used are available from Supplementary table 2.

Health worker knowledge was assessed by means of a supervised self-completed multi-choice questionnaire that was based on 3 clinical case scenarios on HT, DM, and HIV infection. For each scenario, the assessment also looked at five elements: 1) essential diagnostic steps (symptoms, signs, tests); 2) risk factors for each of these 3 disease; 3) complications; 4) treatment regimens; and 5) guidelines for referral. The HIV case scenario was included for comparison, as earlier knowledge tests had indicated that HWs in East Africa had generally good knowledge on HIV case management [17, 18]. Each scenario was worth 10 points, so a HW could score a maximum of 30 points.

The inspection score from the SARA tool and the scores from the health worker clinical knowledge assessment were combined into a single aggregate facility performance score (FPS). The score from the SARA tool was converted to a percentage and scaled to a maximum of 60 points (e.g., a facility that scored 40 on the SARA would have achieved 40/50 = 0.80*60 = 48 points). Similarly, HW knowledge scores for each facility were totalled and converted to a percentage, then scaled to a maximum of 40 points. For example, if there were 5 HWs who accumulated a total of 132 points out of 150 (5 × 30) possible points (equivalent to 88%), the facility received a total of (40*0.88) = 35 points (rounded to the nearest whole number) for the knowledge assessment. The SARA and knowledge assessments points were then combined into the FPS with a maximum score of 100. Using the above example, the FPS would then be 48 + 35 = 83 out of a possible maximum of 100 points.

Additional details on health worker characteristics are available from Supplementary table 1.

### Assessment of quality of patient care and experience (QoCE)

The QoCE assessment comprised five questions assessing whether: 1) the patient was diagnosed correctly; 2) treatment was provided; 3) the patient received health education; 4) the quality of reception and waiting time were acceptable; and 5) the patient was managed according to national guidelines (Table 3). The observations were summarised into a QoCE score. Each question could result in up to 2 points, for a maximum score of 10. The patient was considered to be adequately managed if their QoCE score was ≥ 7/10.

**Table 3:** Data collection sheet to assess quality of patient care and experience (QoCE)

Item	Possible score (circle)	Possible points (circle)	Total points achieved
1. Patient has been diagnosed correctly	No	0	
	Partial evidence^a^	1	
	Correctly diagnosed, supporting evidence was recorded	2	
2. Treatment provided	No evidence	0	
	Partial evidence^a^	1	
	Treatment provided with sufficient drugs and/or guidance provided until next visit	2	
3. Patient received health education	No evidence	0	
	Patient is aware of symptoms, risks, and complications	1	
	Patient is also aware of recommended lifestyle changes	2	
4. Quality of reception and waiting time	Patient had to wait for more than 2 h	0	
	Patient had to wait less than 2 h	1	
	Patient had to wait but received support or health education during waiting time	2	
5. Management according to guidelines	Patient is not being managed according to guidelines	0	
	Patient is being partially managed acc. to guidelines	1	
	Patient is being fully managed acc. to guidelines	2	
	**Total points achieved**		**!__! !__!**
	**Patient adequately managed** **Yes 1 No 2**		**!__!**

^a^Evidence from available records not clear or insufficient to ascertain the correct diagnosis or treatment

### Statistical methods

Data were collected by a team comprising a study clinician or study nurse and 3 field workers, mainly via hand-held tablets using REDCap® version 7.6.3. Data from REDCap® were checked in the field by the clinician or the nurse and were then actively synced or uploaded on to study servers at the end of each day. Facility inspection data were collected on paper checklists and double entered in REDCap®. All data entry from paper sources was overseen by a senior data manager and REDCap® programmer. If necessary, queries were raised and communicated to the field team for verification.

All analyses were conducted in Stata 17.0 (College Station, Texas, USA). Continuous variables were summarised as means and standard deviations (SD), or medians and interquartile ranges (IQR). Categorical variables were summarised with frequency counts and percentages. Characteristics of health workers and patients surveyed in 2020 and 2016 were compared using the Pearson chi-squared statistic with the second-order correction of Rao and Scott to account for the clustered design.

The aggregate FPS was the primary outcome used to assess the impact of the lack of research-driven support on service availability and readiness assessment in 2020. The analysis used methods designed for pair-matched cluster randomised trials, with each HF in 2020 being treated as paired with its observation from 2016. First, the mean aggregate FPS was calculated for each HF in 2016 and in 2020, and log transformed. Then, the difference in log mean scores between 2020 and 2016 was calculated for each HF, and a paired t-test was performed on the pair-wise differences. A similar analysis was done for each component of the FPS: the facility inspection score and the health worker knowledge score.

The association between the aggregate FPS in 2020 and factors that may have influenced service quality was explored using linear regression. These factors included the functionality of patient clubs, whether the HFs had external support such as extra drug supplies from a non-governmental organisation (NGO) or community-based organisation (CBO), HF level, area (rural, peri-urban, or urban), district (Mpigi or Wakiso), and pairing at randomisation (before the start of the original trial). Initial models evaluated each factor individually and adjusted for the FPS in 2016. All variables that were associated with the 2020 score at *p* < 0.20 after adjusting for the 2016 score were included in a multivariable model (the ‘fully adjusted’ model).

The QoCE score, and proportion of adequately managed patients (QoCE ≥ 7), was used to assess the impact of the lack of research-driven support on the quality of patient care in 2020, using the same analysis approach as for the FPS. First, the mean QoCE score and the prevalence of adequately managed patients in each HF in 2016, and in 2020, was calculated and log transformed. Then, the difference in log mean scores, and in log prevalence, between 2020 and 2016 was calculated for each HF, and a paired t-test was performed on the pair-wise differences.

The association between QoCE scores in 2020 and factors that may have influenced quality of care was explored in an individual-level analysis using a Tobit regression with random effects to account for the correlation of multiple observations within HF. Since the QoCE score is bounded by a maximum of 10, ordinary linear regression can lead to biased standard errors. Therefore, Tobit regression is used to reduce the bias in the estimation of standard errors when the outcome is censored (bounded) [19]. These factors included the patients’ age, gender, the functionality of patient clubs, whether the HFs had external support such as extra drug supplies from a non-governmental organisation (NGO) or community-based organisation (CBO), HF level, area (rural, peri-urban, or urban), district (Mpigi or Wakiso), and pairing at randomisation. Initial regression models evaluated each factor individually and when adjusted for the facility mean QoCE score in 2016 a priori. All variables that were associated with the QoCE score in 2020 at *p* < 0.20 after adjusting for the mean score in 2016 were included in a multivariable model.

## Results

### Service availability and readiness

All 22 health facilities were inspected using the SARA tool. A total of 91 HWs from the 22 facilities were assessed on the clinical knowledge test, compared with 131 from the EACDRP trial in 2016. The lower number in 2020 was due to COVID restrictions and absenteeism. 60 HWs (66%) surveyed had also completed the test in 2016. The age distribution across the two years was similar with a mean (SD) age of 37.0 (7.9) in 2020 compared with 36.6 (9.6) in 2016 (*p* = 0.01). A slightly lower proportion of respondents in 2020 were female than in 2016 (65% vs 70%, respectively, *p* = 0.11), and a larger proportion were clinicians 27% in 2020 vs 17% in 2016), although the difference was not statistically significant (*p* = 0.29). Most respondents were either nurses, midwives, or nursing aides (67% in 2020 and 76% in 2016). In 2020, 47% of HWs were from HCIIIs and 28% were from HCIIs, compared with 60% and 18% in 2016, respectively, while 25% of HWs in 2020 and 22% in 2016 were from HCIVs (*p* < 0.01) (Table 7).

The mean aggregate FPS from the facility inspection and clinical knowledge assessments in 2020 was lower than in 2016: 70.2 (95%CI = 66.0–74.5) compared with 74.8 (95%CI = 71.3–78.3), respectively (Table 4). However, there was no evidence of a significant difference (*p* = 0.18).

**Table 4:** Summary of facility performance scores (FPS) and five elements of service availability and readiness (SAR)

	**Mean score (95% CI)**	
	**2016**	**2020**
**Overall**		
		*P* = 0.18^1^
Overall FPS aggregate (inclusive of knowledge assessment scores)^a^	74.8 (71.3–78.3)	70.2 (66.0–74.5)
**Individual elements**^b^		
		*P* < 0.001
Availability & functionality of essential equipment /consumables	7.59 (7.27, 7.91)	5.56 (4.98, 6.14)
		*P* < 0.001
Availability of essential drugs and other consumables	8.28 (6.57, 9.99)	3.71 (2.46, 4.97)
		*P* = 0.001
Quality of records on case management of HT and DM	9.09 (8.68, 9.50)	9.77 (9.54, 10.01)
		*P* < 0.001
Utilisation of health facility by HT & DM patients	8.00 (7.33, 8.67)	3.00 (1.82, 4.18)
		*P* < 0.001
Evidence of preventive activities for HT & DM	7.18 (6.56, 7.80)	3.77 (2.87–4.67)

^1^*p*-values obtained from paired t-test of difference in log-transformed mean in each HF

^a^Includes both scaled-up health worker knowledge assessment (max. 40 points) and SAR scores (max. 60 points) out of a maximum 100 points

^b^Score out of 10 points for each element

In evaluating the separate components of the FPS score, across the 5 elements of the modified SARA tool for facility inspection, we observed a decline in mean scores of 4 of the components, ranging from 2 to 5 points: the *availability of essential equipment, essential drugs, utilisation of NCD treatment services* and *preventive services* (Table 4). The *utilisation of NCD services for HT and DM* was most adversely impacted, with a mean score of 3.0 (95%CI 1.8, 4.2) in 2020 compared with 8.0 (95%CI = 7.3, 8.7) in 2016 (*p* < 0.001). In contrast, the mean score for the *quality of records* showed an improvement: 9.8 (95%CI = 9.5, 10.0) in 2020 vs 9.1 (95%CI = 8.7, 9.5) in 2016 (*p* = 0.001).

Mean health worker knowledge scores were slightly higher in 2020 than 2016: 26.8 (95%CI = 26.1, 27.5) vs 26.0 (95%CI = 25.3, 26.7), although the difference was not significant (*p* = 0.11) (Table 5). There was some evidence that mean knowledge scores for HIV were higher in 2020 than in 2016 (8.5 vs 8.2, respectively; *p* = 0.08), but no evidence that knowledge scores differed for the other disease areas or across HF levels (Table 5).

**Table 5:** Summary of health worker knowledge assessment

Health worker knowledge assessment	2016 mean (95% CI)	2020 mean (95% CI)	*P*-value*
***Overall & by health facility level (out of 30 maximally possible points)***			
*** Overall***	***N* = *131***	***N* = *91***	0.11
	26.0 (25.3–26.7)	26.8 (26.1–27.5)	
*** HCIIs***	***N* = *24***	***N* = *25***	0.43
	25.1 (23.7–26.5)	25.9 (24.6–27.1)	
*** HCIIIs***	***N* = *78***	***N* = *43***	0.22
	26.8 (26.0–27.6)	27.5 (26.5–28.4)	
*** HCIVs***	***N* = *28***	***N* = *23***	0.49
	26.1 (21.9–30.2)	27.3 (25.1–29.5)	
***By each disease component (out of 10 maximally possible points)***			
*** Hypertension***	9.1 (8.8, 9.4)	9.3 (9.1, 9.6)	0.22
*** Diabetes***	8.8 (8.4, 9.1)	8.9 (8.6, 9.2)	0.42
*** HIV***	8.2 (7.9, 8.4)	8.5 (8.2, 8.8)	0.08

^*^p-values obtained from paired t-test of difference in log-transformed mean in each HF

The FPS in 2020 was inversely associated with FPS in 2016, with mean FPS in 2020 decreasing by 0.45 points (95%CI -0.77, 0.12; *p* < 0.01) for every point increase in the 2016 score. After adjusting for the FPS in 2016, there was strong evidence that the FPS in 2020 was associated with location of the HF (*p* < 0.01) (Table 6). The mean FPS was 10.9 points lower in HFs located in rural areas (95%CI = -19.9, -1.91) and 20.6 points lower in those located in urban (95% CI -32.8, -8.42) compared with peri-urban areas. There was borderline association with a HF’s patient club functionality (*p* = 0.05) with moderate and low functionality associated with a mean FPS that was 5.62 (95%CI = -13.3, 2.00) and 6.82 (95%CI = -14.1, 0.47) points lower, respectively, than patient clubs with high functionality. There was some evidence of an association between FPS and facility level (*p* = 0.08), with mean FPS scores 1.12 points higher (95%CI = -5.64, 7.89) in HCIIIs than in HCIIs and 8.90 points higher (95%CI = -0.65, 18.5) in HCIVs than in HCIIs. There was also weak evidence that the FPS was lower in Mpigi than in Wakiso (-4.57, 95%CI = -10.7, -1.59, *p* = 0.11). There was no evidence of an association with post-trial external support received (e.g., from NGOs) (*p* = 0.22) or pairing at randomisation (0.19) (Table 6).

**Table 6:** Unadjusted and adjusted analysis of the effect of independent factors on facility performance (FPS)

Variable	Level	Model 1 Coefficient, (95% CI)	Model 2^a^ Coefficient, (95%CI)	Model 3^b^ Coefficient, (95%CI)
**Mean FPS score in 2016**		*P* < 0.01	*P* < 0.01	*P* < 0.01
		-0.45 (-0.77, 0.12)	-0.45 (-0.77, -0.12)	-0.55 (-0.89, -0.21)
**Patient club functionality**		*P* = 0.26	*P* = 0.05	*P* = 0.09
	High	Ref	Ref	Ref
	Moderate	-4.98 (-14.30, 4.34)	-5.62 (-13.25, 2.00)	-6.30 (-14.96, 2.35)
	Low or none	-5.11 (-13.94, 3.72)	-6.82 (-14.12, 0.47)	-4.96 (-12.97, 3.06)
**Other external support received by HF**^**c**^		*P* = 0.44	*P* = 0.22	-
	No	Ref	Ref	-
	Yes	2.74 (-4.52, 10.01)	3.43 (-2.75, 9.62)	-
**Facility level**		*P* = 0.70	*P* = 0.08	*P* = 0.81
	HCIIs	Ref	Ref	Ref
	HCIIIs	-2.69 (-10.34, 4.95)	1.12 (-5.64, 7.89)	-0.93 (-8.33, 6.47)
	HCIVs	4.56 (-6.54, 15.66)	8.90 (-0.65, 18.46)	3.02 (-13.55, 17.93)
**Area**		*P* = 0.10	*P = *0.01	*P* = 0.23
	Peri-urban	Ref	Ref	Ref
	Rural	-5.84 (-18.02, 6.34)	-10.90 (-19.90, -1.91)	-6.22 (-24.65, 12.21)
	Urban	-13.02 (-29.36, 3.32)	-20.59 (-32.76, -8.42)	-12.91 (-37.36, 11.54)
**District**		*P* = 0.15	*P* = 0.11	*P* = 0.19
	Mpigi	Ref	Ref	Ref
	Wakiso	-5.13 (-12.28, 2.02)	-4.57 (-10.73, 1.59)	-3.03 (-9.54, 3.48)
**Pairing at randomisation**		*P* = 0.31	*P* = 0.19	*P* = 0.60
	Singleton	Ref	Ref	Ref
	Paired or Referral	3.53 (-3.53, 10.59)	3.59 (-2.44, 9.62)	-1.21 (-5.58, 7.99)

^a^Adjusted for baseline FPS scores in 2016

^b^Adjusted for baseline FPS, patient club functionality, facility level, area, district and pairing at randomisation

^c^Post trial external support includes any supplementary drugs or other support that a HF received between 2016–20 from an NGO or CBO excluding its patient club

In the final adjusted model, after adjusting for patient club functionality, facility level, HF area, district and pairing at randomisation, the mean FPS in 2020 decreased by 0.55 points (95% CI -0.89, -0.21) for every point increase in the 2016 score (*p* < 0.01). None of the other factors was found to be an independent predictor of FPS but club functionality showed a weak association (*p* = 0.09) (Table 6).

### Quality of patient care and experience

QoCE assessments were available from interviews with 332 patients in 2020 and 88 patients from 2016. The patients in 2020 were older than in 2016 (mean (95%CI) = 60.1 (58.5, 61.8) vs 55.9 (53.6, 58.1)), respectively (*p* < 0.01) but the gender distribution was similar across both groups (71% female in 2020 vs 65% in 2016, *p* = 0.22). Patient distribution across HF levels was not different (*p* = 0.86). More than half of patients (53%) in 2020 said they were employed compared to 29% in 2016 respectively while 40% in 2020 considered themselves to be homemakers compared to 47% in 2016. Only 7% were either unemployed or retired or reported belonging to other categories in 2020 compared to about a quarter (24%) in 2016 (*p* < 0.01).

There was good evidence that the mean QoCE score was lower in 2020 than in 2016 (8.72 vs 9.45, respectively, *p* = 0.02) (Table 7). The proportion of *adequately managed patients* was also lower in 2020 (88.2% vs 95.5%), but the difference was not significant (*p* = 0.20). When stratified by HF level, the largest decrease in QoCE scores was seen in HCIIs (a mean decrease of 0.91 points, 95% CI = -1.82, -0.003; not shown).

**Table 7:** Summary of patient quality of care and experience (QoCE) assessment

Variable	2016 *N* = 88	2020 *N* = 332	*p*-value^1^
**Gender**^a^			
*Women*	56 (65)	237 (71)	0.22
*Men*	30 (35)	95 (29)	
**Age**			
*Mean (95%CI)*	55.9 (53.6, 58.2)	60.1 (58.5, 61.7)	< 0.01
**Health facility level**			
*HCIIs*	40 (45)	145 (44)	< 0.01
*HCIIIs*	36 (41)	134 (40)	
*HCIVs*	12 (14)	53 (16)	
**Occupation**			
*Employed*	26 (29)	176 (53)	< 0.01
*Homemaker*	41 (47)	132 (40)	
*Unemployed/Retired/Other*	21 (24)	24 (7)	
**Overall QoCE score**			
*Mean score (95% CI)*	9.45 (9.05–9.86)	8.72 (8.36–9.08)	0.02
*Proportion adequately managed*	95.5%	89.2%	0.24
**QoCE score by health facility level**			
***HCIIs ***			
*Mean score (95% CI)*	9.78 (9.48–10.07)	8.87 (8.15–9.58)	0.05
*Proportion adequately managed*	100%	88.0%	0.34
***HCIIIs ***			
*Mean score (95% CI)*	9.12 (8.31–9.94)	8.41 (8.05–8.76)	0.14
*Proportion adequately managed*	90.0%	87.0%	0.74
***HCIVs***			
﻿ *Mean score (95% CI)*	9.58 (9.12–10.04)	9.30 (8.70–9.89)	0.61
*Proportion adequately managed*	100%	96.4%	0.64

^1^*P*-values from paired t-test of log-transformed facility-level summaries (mean score or proportion of adequately managed patients)

^a^2 patients missing gender in 2016

There was no evidence that mean QoCE scores in 2016 were associated with QoCE scores in 2020 (*p* = 0.71). After adjusting for QoCE scores in 2016, there was some evidence that scores in 2020 were associated with patient gender, external support (excluding patient club support), district, and patient club functionality (Table 8). Mean QoCE scores in 2020 were 0.43 points higher (95%CI = -0.08, 0.95; *p* = 0.10) in women than men, 1.35 points higher (95%CI = -0.28, 2.41; *p* = 0.02) in HFs with external support than those without, and 1.04 points higher (95%CI = -0.09, 2.17; *p* = 0.08) in Wakiso than Mpigi district. Mean QoCE scores in 2020 were lower in HFs with low or no patient club functionality compared with those with high functionality (-0.92, 95%CI = -2.19, 0.36; *p* = 0.05). After adjusting for QoCE scores in 2016, gender, patient club functionality, external support, facility level, area, and district, only parent district remained an independent predictor of QoCE scores in 2020, with HFs in Wakiso having scores 1.21 points higher (95%CI = 0.25, 2.18; *p* = 0.02) than Mpigi.

**Table 8:** Unadjusted and adjusted analysis of the effect of independent factors on quality of patient care and experience (QoCE) using Tobit regression

Variable	Model 1	Model 2^a^	Model 3^b^
	**Coefficient (95%CI)**	**Coefficient (95%CI)**	**Coefficient (95%CI)**
**Mean QoCE score in 2016**	*P* = 0.71	*P* = 0.71	*P* = 0.75
	0.13 (-0.53, 0.78)	0.13 (-0.53, 0.78)	0.08 (-0.42, 0.58)
**Age**	*P* = 0.48	*P* = 0.49	-
	-0.07 (-0.25, 0.12)	-0.07 (-0.25, 0.12)	
**Gender**	*P* = 0.10	*P* = 0.10	*P* = 0.13
Male	Ref	Ref	Ref
Female	0.43 (-0.08, 0.95)	0.43 (-0.08, 0.95)	0.40 (-0.12, 0.91)
**Patient club functionality**	*P* = 0.05	*P* = 0.05	*P* = 0.87
High	Ref	Ref	Ref
Moderate	0.71 (-0.62, 2.04)	0.68 (-0.65, 2.02)	0.31 (-0.88, 1.51)
Low or none	-0.90 (-2.18, 0.37)	-0.92 (-2.19, 0.36)	0.09 (-1.26, 1.43)
**External support**^**c**^	*P* = 0.02	*P* = 0.02	*P* = 0.20
No	Ref	Ref	Ref
Yes	1.32 (0.25, 2.39)	1.35 (0.28, 2.41)	0.77 (-0.38, 1.92)
**Facility level**	*P* = 0.15	*P* = 0.15	*P* = 0.64
HC IIs	Ref	Ref	Ref
HC IIIs	-0.94 (-2.12, 0.23)	-0.99 (-2.24, 0.25)	-0.53 (-1.61, 0.56)
HC IVs	0.52 (-1.15, 2.20)	0.51 (-1.17, 2.19)	-0.34 (-2.62, 1.94)
**Area**	*P* = 0.13	*P* = 0.13	*P* = 0.37
Peri-urban	Ref	Ref	Ref
Rural	-1.40 (-3.28, 0.47)	-1.42 (-3.28, 0.45)	-1.28 (-4.00, 1.45)
Urban	-2.84 (-5.54, -0.14)	-2.88 (-5.57, -0.19)	-2.60 (-6.37, 1.18)
**District**	*P* = 0.09	*P* = 0.08	*P* = 0.02
Mpigi	Ref	Ref	Ref
Wakiso	1.03 (-0.10, 2.16)	1.04 (-0.09, 2.17)	1.21 (0.25, 2.18)
**Pairing at randomisation**	*P* = 0.64	*P* = 0.66	-
Singleton	Ref	Ref	
Paired or Referral	-0.28 (-1.45, 0.90)	-0.26 (-1.44, 0.91)	

^a^Adjusted for mean QoCE score in 2016

^b^Adjusted for mean QoCE score in 2016, gender, club functionality, external support, facility level, area, and district

^c^Post trial external support includes any supplementary drugs or other support that a HF received between 2016–20 from an NGO or CBO excluding its patient club

## Discussion

We defined facility performance as having two inputs i) health worker clinical knowledge and ii) the facility inspection with five constituent elements of service availability and readiness.

Overall, health worker knowledge was well preserved, and knowledge scores were similar across all facility levels whether by subject matter or the 3 disease case scenario questions. This indicates that over the post-trial period of 4 years, despite any decay or shortcomings in support supervision or motivation or even underutilisation of the facility; the NCD case management competence of HWs were largely sustained. Only 66% of HWs interviewed in 2020 took part in the assessment in 2016 which suggests that NCD related knowledge among new staff members was also adequate. This finding was also reinforced by a similar finding on adequate quality of care and experience which remained unchanged over this same period.

Health worker knowledge is usually measured as a pre- and post-training assessment we have had the benefit of assessing it at least 4 years after the intervention. Interestingly, most studies in SSA that have assessed task shifting with nurse-led NCD management, have shown good knowledge retention in using a qualitative framework approach [20] or the bundled education and support with text (BEST) method [21]. However, our study like others in Kenya and South Africa [22, 23], also demonstrates that a protocol-driven or clinical knowledge test approach based on national guidelines is essentially comparable.

Despite the decline in most of the elements of service availability and readiness over the period 2016–2020, there was no evidence of difference in overall facility performance owing to the strong performance of HWs on the clinical knowledge test. Of the five constituent elements of SAR, only the quality of records was preserved while the utilisation of NCD services and evidence of preventive activities declined most strongly. The availability of essential drugs and to a lesser extent the availability of essential equipment and consumables also declined, but less steeply. This suggests that the support obtained from functional patient clubs may have alleviated the performance decline to some extent, through the replenishment of consumables, repair or replacement of simple equipment and the direct purchase of essential medicines. However, with regards to the availability of essential medicines, the worst performing HFs only scored < 2 points out of possible 10 in 2020 (not shown), indicating that some HFs were more severely impacted by inconsistent drug supplies than others, and also had little to no patient club support.

The utilisation of primary care services at public facilities usually reflects the availability of essential medicines and other consumables [24–26], and therefore the observed decline in utilisation was expected. This decline is most likely also a result of a reduction in screening efforts, consistent with our finding that preventive activities in 2020 occurred much less frequently than in 2016. This in turn implies that only a few new NCD cases were actively detected and put into NCD care.

The association of FPS with patient club functionality (*p* = 0.05) and facility level (*p* = 0.08) was borderline. District location (Mpigi vs Wakiso) (*p* = 0.19) and the peri-urban vs rural or urban location of a health facility (0.23) did not affect service availability and readiness. None of them predicted facility performance independently despite a weak association with patient club functionality (0.09). However, adjusting for 2016 scores, rural and urban HFs mean scores in 2020 were 11 and 21 points lower than peri-urban HFs (*p* < 0.01) respectively, perhaps a chance finding. Our findings are reminiscent of a recent study from southern Nigeria which found that service readiness increased with the presence of some power sources (electricity, generators, batteries and solar), but was lower among lower-level units that did not have this support. Travel time to headquarters and rural facilities also significantly reduced indices of equipment availability [27]. We did not assess the effect of electricity or power sources on facility performance in this study. However, lower-level facilities in Uganda have at least one power source from either solar panels or public electricity [28]. The availability and functionality of the solar equipment or electricity system lies in the docket of the parent district; however, district location was not found to affect facility performance but was found to be an independent predictor of service quality instead.

Our findings also differ from the Nigerian study with regards to the urban health facility location, which in Uganda we speculate may be due to an intervening local administrative level at the municipality or local town council that usually distorts the district office’s direct influence. A district health officer or their team may not exercise the same direct supervisory oversight and authority over those units within a semi-autonomous local municipality as those outside because these usually report to the local municipality health officer instead. This gap often leads to poor district support in terms of consistent supervisory oversight and timely medical supplies or replenishments especially where the municipality or local authority administration is weak. Public health facilities in urban areas are often poorly utilised as urban-dwelling patients have more health care options such as services from commercial drug shops and private clinics [29]. Public HFs in Uganda are also generally shunned because of inconsistent drug supplies, health worker absenteeism and poor overall supervision [30].

The weak association of FPS with HF level in the initial models where higher-level units performed up to 9-points better than HCIIs (or HCIIIs) can be partly explained by the fact that these facilities are referral clinics and have more consistent drug supplies. They also usually have stronger patient clubs.

The quality of patient care and experience showed a statistically significant decline between 2016 and 2020 (*p* < 0.02). The proportion of patients receiving adequate quality of care in line with national guidelines also declined but not significantly so (*p* = 0.24). A U-shaped relationship between service quality and patient volume has been previously described by a service quality assessment in Ethiopia which indicated that service quality increases until a peak patient volume of 90 patients per day and then decreases [31]. Whereas we did not measure patient volume directly as related to service quality, we did measure service utilisation under facility performance which had dropped in 2020 to less than half of that observed in 2016 (8 vs 3-points; *p* > 0.001). It can be argued that service utilisation in 2020 was only about half of that seen when the intervention had reached its peak optimisation in 2016. This might explain our mixed findings; we would speculate that indeed some HFs had in fact optimised, and also reached their patient volume thresholds with a subsequent dip in service quality thereafter. Whilst other HFs might still be optimising or re-optimising service quality below their patient volume thresholds currently.

The quality of patient care was strongly associated with parent district (*p* = 0.02). Wakiso district was found to have 1.2-point higher increase in mean QoCE than Mpigi, this association appeared to stronger after controlling for all other factors. Wakiso district geographically encircles the capital city of Uganda, Kampala, and as such benefits from the better road network that radiates from the capital city in a variety of directions. This has the double effect of ensuring quicker and more regular replenishment of essential medical supplies to most of Wakiso district as well as more regular support supervision due to easier access to remote district HFs. However, this could also be a chance finding as logistical support to NCD services did not seem to differ that much between the two districts.

Regarding provider or HS quality of care studies on NCDs in SSA, one study in Lesotho within HIV clinics [32] found that about a third of patients did not have records on NCD outcomes. The main barriers to care were equipment shortage or disrepair and staff shortage which affected the organisational structure for NCD care while inadequate screening for NCDs, poor scheduling and inadequate patient education affected treatment processes [32]. This is not very different from what we found regarding the challenges to facility performance on very similar 4 of the 5 elements of service readiness. However, in our study one element i.e., the quality of records was adequately preserved over the 4 years.

### Strengths

The EACDRP intervention project had provided a strong and effective NCD service intervention with fully optimised elements against which it was easy to study potential changes in service readiness or service quality over time. For example, record keeping had been optimised in intervention HFs during the trial, data quality was high in both consecutive surveys. This applied to both routine data collection tools and records e.g., patient registers.

Identical and standardised study tools were used at both time points. To avoid observed bias, care was taken to ensure that observed data collection in 2020 did not involve any staff member from the original intervention (implementation) team. Also, instead, we recruited staff from the original evaluation team, and this helped to ensure comparability of the data sets generated at the two time points. The restricted sampling technique used for the QoCE assessment, allowed us to sample up to 20 eligible patients who were invited without replacement for defaulting, and also to ensure appropriate representation of men and women and patient groups (i.e., for DM and HT). This also helped increase the power of the study.

Lastly, most post-intervention evaluations in a research or programmatic setting are usually done after 2 or 5 years, the 2020 survey was conducted about 4 years after the end of the intervention trial. This interval was sufficiently long to study mid- to long-term sustainability and durability of intervention effects.

### Limitations

We were not able to collect data on service readiness and quality of care at any point of time between 2016 and 2020. We, therefore, could not assess whether the observed declines had occurred earlier or later, and whether the decline followed a linear pattern or occurred at certain points in time. Because there were no major changes in policy, infrastructure, or human resource or staff attrition in the assessed intervention facilities over this period; we assume the deterioration occurred slowly.

We also cannot ascertain whether any effect of the intervention may have in fact optimised after 2016, and whether any positive effects observed in 2020 reflected that subsequent optimisation rather than intervention durability. However, the 4-year gap between assessments is likely to be a long enough period to hone out only the durable post-trial effects.

As this study focused on evaluating sustainability in facilities that received the intervention, we did not evaluate the former control facilities for comparison; therefore, it is possible that our findings were a result of other external influences that might have alleviated a possible decline of SAR and/or QoCE, rather than of the durability of the intervention. However, there were no major changes in health policy, health facility management or community initiatives during this period, and we are not aware of any other (e.g., NGO-related) health service efforts to strengthen health service performance, neither among the former intervention nor the former control facilities. Although all primary care centres in Uganda participate in ongoing efforts of the national NCD control programme to strengthen the response to NCDs, the effect of these efforts has been limited: for example, the proportion of health workers trained “during the last year” was 23% in 2020 and 39% in 2016, and there was little difference in having “received any NCD training” (68% in 2020 vs. 65% in 2016).

Our sample size of health workers and patients in 2016 was fixed; therefore, our ability to increase the power of the study was limited. However, where possible, we increased the number of participants in the 2020 survey so that we had reasonable power (> 80%) to detect important changes in service readiness or quality of care.

Both health workers and patients who participated in the MeLoHanD study represent clusters of participants: it can be assumed that individuals from the same HF were likely to be more similar with regards to the variables that we determined in this study than a random selection of independent individuals. We accounted for this clustered design in the statistical analysis.

However, there were some notable differences in the study populations between the two surveys e.g., there were fewer health workers at HCIIs in 2016 than in 2020 (18% vs 28%) and more at HCIIIs (60% vs 47%) which could mean that HCIIs were over-represented or HCIIIs were under-represented in the 2020 health worker sample. This could be partly explained by the fewer number of health workers that were available for survey in 2020 due to COVID restrictions or related absenteeism. In spite of this, there was generally a more equitable spread in 2020.

## Conclusions

The clinical knowledge of HWs was sustained over 4 years after the end of the intervention project. This suggests that within similar primary care settings in LMICs, training effects resulting from the original intervention are likely to be maintained for some extended period, even as service availability and readiness decline in the absence of funding support. Nevertheless, we recommend that refresher training of health workers should be routinely provided within a public health care system until such a time when other elements of service readiness are sufficiently optimised.

As expected, overall facility performance was negatively affected because of the interrupted supplies and a decline in regular supervision. Some intervention components declined more strongly in urban or rural than peri-urban settings; and we speculate that this depended on administrative impediments and supervision. Lastly, the availability of functioning patient clubs seemed to have a positive effect with regards to service availability and readiness.

Surprisingly, although the overall quality of care received and experienced by HT and DM patients had declined, the proportion of those receiving adequate care according to national guidelines had not substantially changed even 4 years after the end of funding support to the intervention. District oversight was important to maintaining service quality.

More research in similar primary care settings is needed to clarify the role of well-functioning patient clubs for the sustainability of NCD care: what is their mean time-to-full optimisation and how and when do they begin to affect the continuity of NCD or other chronic care services in the absence of any funding support.

Sustainability or durability of an NCD intervention in similar primary care settings may remain achievable despite the funding instability and logistical challenges that follow withdrawal of research or programme support. Early during an intervention trial, health system managers and researchers should jointly plan how to sustain the intervention beyond the end of the project if the trial demonstrates its effectiveness.

### Supplementary Information

**Additional file 1: Supplementary table 1.** Health worker survey – characteristics of HWs in 2016 and 2020.

**Additional file 2: Supplementary table 2.**- Facility inspection - distribution of the constituents of the elements of service availability and readiness (SAR).

## Data Availability

The datasets that support these findings are available from London School of Hygiene and Tropical Medicine (LSHTM) and MRC/UVRI and LSHTM Uganda Research Unit (MUL), but restrictions apply to the availability of these data, which were used under licence for the current study, and so are not publicly available. However, data are available from the authors (David Katende) upon reasonable request and with permission of both LSHTM and MUL. Contact person: Ayoub Kakande Email: Ayoub.Kakande@mrcuganda.org.

## Abbreviations

CBO: Community Based Organisation
CI: Confidence Interval (usually 95%)
DHO: District Health Office(r)
DM: Diabetes mellitus
EACDRP: EACDRP – East African Chronic Disease Research Project
FPS: Facility Performance Score(s)
HC(s): Health Centre(s) Is, IIs, IIIs, IVs, termed HCs of level 1, 2, 3, 4)
HF(s): Health Facility (Health Facilities)
HIV: Human Immuno-deficiency Virus
HS: Health system(s)
HT: Hypertension
HW(s): Health worker(s) / Healthcare worker(s)
IQR: Interquartile range
LSHTM: London School of Hygiene and Tropical Medicine
LMICs: Low- and middle- income countries
MeLoHanD: Medium-to-Long term sustainability to improve management of Hypertension and Diabetes within the primary care setting in Uganda
MoH: Ministry of Health (Uganda)
MRC: Medical Research Council
NCD(s): Non-communicable disease(s)
NGO: Non-governmental Organisation
UVRI: Uganda Virus Research Institute
SAR(A): Service Availability and Readiness (Assessment)
SSA: Sub-Saharan Africa
SD: Standard deviation
QoC(E): Quality of Care (and Experience)

## References

[1] Boutayeb A, Boutayeb S (2005). The burden of non communicable diseases in developing countries. Int J Equity Health.

[2] Collaborators GBDRF (2020). Global burden of 87 risk factors in 204 countries and territories, 1990–2019: a systematic analysis for the Global Burden of Disease Study 2019. Lancet..

[3] Hendriks ME, Wit FW, Roos MT, Brewster LM, Akande TM, de Beer IH (2012). Hypertension in sub-Saharan Africa: cross-sectional surveys in four rural and urban communities. PLoS ONE.

[4] Kavishe B, Biraro S, Baisley K, Vanobberghen F, Kapiga S, Munderi P (2015). High prevalence of hypertension and of risk factors for non-communicable diseases (NCDs): a population based cross-sectional survey of NCDS and HIV infection in Northwestern Tanzania and Southern Uganda. BMC Med.

[5] Wesonga R, Guwatudde D, Bahendeka SK, Mutungi G, Nabugoomu F, Muwonge J (2016). Burden of cumulative risk factors associated with non-communicable diseases among adults in Uganda: evidence from a national baseline survey. Int J Equity Health.

[6] UNGASS. Political Declaration of the High-level Meeting of the General Assembly on the Prevention and Control of Non-communicable Diseases United Nations General Assembly, 66th session. 2011. Available from: http://www.un.org/en/ga/ncdmeeting. Accessed 12 May 2023.

[7] Schwartz JI, Dunkle A, Akiteng AR, Birabwa-Male D, Kagimu R, Mondo CK (2015). Towards reframing health service delivery in Uganda: the Uganda Initiative for Integrated Management of Non-Communicable Diseases. Glob Health Action.

[8] Kapiga S, Munderi P, Katende D, Kavishe B, Lubega G, Nsanya M, et al. Improving the health systems response to chronic non-communicable diseases at primary care facilities in Uganda and Tanzania: Results from a cluster-randomised controlled trial. . BMC Med. 2022. (Under review)

[9] Shediac-Rizkallah MC, Bone LR (1998). Planning for the sustainability of community-based health programs: conceptual frameworks and future directions for research, practice and policy. Health Educ Res.

[10] UBOS. Uganda Demographic and Health Survey 2016 Key Indicators Report. 2017. Available from: https://www.ubos.org/wp-content/uploads/publications/03_2018Uganda_DHS_2016_KIR.pdf. Accessed 2 Feb 2023.

[11] MOH. Uganda Health Sector Strategic Plan III (HSSP III) 2010/11–2014/15 Ministry of Health U. 2010. Available from: https://www.health.go.ug/docs/HSSP_III_2010.pdf. Accessed 8 Dec 2022.

[12] MOH. Uganda Clinical Guidelines 2012. National guidelines for management of common conditions. Ministry of Health U. 2012. Available from: https://health.go.ug/docs/UCG_2012.pdf. Accessed 9 Dec 2022.

[13] Ivers NM, Halperin IJ, Barnsley J, Grimshaw JM, Shah BR, Tu K (2012). Allocation techniques for balance at baseline in cluster randomized trials: a methodological review. Trials.

[14] UBOS. The Uganda National Household Survey 2009/10: Report on the Socio-Economic Module. Abridged Report. Kampala, Uganda, UBOS. 2010. Available from: https://www.ubos.org/wp-content/uploads/publications/03_2018UNHS_2009_2010_socio-economic_Report.pdf. Accessed 12 May 2023.

[15] UBOS. Uganda Demographic and Health Survey 2011. Kampala, Uganda: UBOS and Calverton, Maryland: ICF International Inc. 2012. Available from: https://dhsprogram.com/pubs/pdf/fr264/fr264.pdf. Accessed 12 May 2023.

[16] WHO. Service Availability and Readiness Assessment (SARA): An annual monitoring system for service delivery (Reference Manual) 2013. Available from: https://apps.who.int/iris/bitstream/handle/10665/104075/WHO_HIS_HSI_RME_2013_1_eng.pdf. Accessed 12 May 2023.

[17] Katende D, Mutungi G, Baisley K, Biraro S, Ikoona E, Peck R (2015). Readiness of Ugandan health services for the management of outpatients with chronic diseases. Trop Med Int Health.

[18] Peck R, Mghamba J, Vanobberghen F, Kavishe B, Rugarabamu V, Smeeth L (2014). Preparedness of Tanzanian health facilities for outpatient primary care of hypertension and diabetes: a cross-sectional survey. Lancet Glob Health.

[19] Twisk J, Rijmen F (2009). Longitudinal tobit regression: a new approach to analyze outcome variables with floor or ceiling effects. J Clin Epidemiol.

[20] Frieden M, Zamba B, Mukumbi N, Mafaune PT, Makumbe B, Irungu E (2020). Setting up a nurse-led model of care for management of hypertension and diabetes mellitus in a high HIV prevalence context in rural Zimbabwe: a descriptive study. BMC Health Serv Res.

[21] Nanyonga RC, Spies LA, Nakaggwa F (2022). The effectiveness of nurse-led group interventions on hypertension lifestyle management: A mixed method study. J Nurs Scholarsh.

[22] Some D, Edwards JK, Reid T, Van den Bergh R, Kosgei RJ, Wilkinson E (2016). Task Shifting the Management of Non-Communicable Diseases to Nurses in Kibera, Kenya: Does It Work?. PLoS ONE.

[23] Coleman R, Gill G, Wilkinson D (1998). Noncommunicable disease management in resource-poor settings: a primary care model from rural South Africa. Bull World Health Organ.

[24] Zakumumpa H, Kiweewa FM, Khuluza F, Kitutu FE (2019). "The number of clients is increasing but the supplies are reducing": provider strategies for responding to chronic antiretroviral (ARV) medicines stock-outs in resource-limited settings: a qualitative study from Uganda. BMC Health Serv Res.

[25] Umlauf R, Park SJ (2018). Stock-outs! Improvisations and processes of infrastructuring in Uganda's HIV/Aids and malaria programmes. Glob Public Health.

[26] Raja S, Wilbur S, Blackburn S. Uganda logistics systems for public health commodities: an assessment report. 2000. https://pdf.usaid.gov/pdf_docs/pnack537.pdf. Accessed 12 May 2023.

[27] Oyekale AS (2017). Assessment of primary health care facilities' service readiness in Nigeria. BMC Health Serv Res.

[28] Ministry of HU, Macro Macro. Uganda Service Provision Assessment Survey 2007. Kampala, Uganda: Ministry of Health/Uganda and Macro International; 2008. http://dhsprogram.com/pubs/pdf/SPA13/SPA13.pdf. Accessed 12 May 2023.

[29] Konde-Lule J, Gitta SN, Lindfors A, Okuonzi S, Onama VO, Forsberg BC (2010). Private and public health care in rural areas of Uganda. BMC Int Health Hum Rights.

[30] Musoke D, Boynton P, Butler C, Musoke MB (2014). Health seeking behaviour and challenges in utilising health facilities in Wakiso district. Uganda Afr Health Sci.

[31] Arsenault C, Yakob B, Tilahun T, Nigatu TG, Dinsa G, Woldie M (2021). Patient volume and quality of primary care in Ethiopia: findings from the routine health information system and the 2014 Service Provision Assessment survey. BMC Health Serv Res.

[32] Mugomeri E, Khama P, Seshea PC, Bekele B, Mojai S, Maibvise C (2017). The occurrence and quality of care of non-communicable diseases in people living with HIV in Maseru, Lesotho: a mixed-methods study. HIV AIDS Rev.

