# Supplementary material for: Medium-to-long term sustainability of a health systems intervention to improve service readiness and quality of non-communicable disease (NCD) patient care and experience at primary care settings in Uganda
Source: BMC Health Serv Res. 2023 Sep 22;23:1022. doi: 10.1186/s12913-023-09983-7 (PMC10514956; doi:10.1186/s12913-023-09983-7)
Supplement: Supplementary file 1 — Additional file 1: Supplementary table 1. Health worker survey – characteristics of HWs in 2016 and 2020. [file 12913_2023_9983_MOESM1_ESM.docx]

Supplementary table 1- Health worker survey – characteristics of HWs in 2016 and 2020

| **Variable** |  | **2016 n (%) (N=131)** | **2020 n (%) (N=91)** | **p-value^1^** |
| --- | --- | --- | --- | --- |
| **Gender** | Female | 92 (70) | 59 (65) | 0.11 |
|  | Male | 31 (24) | 32 (35) |  |
|  | *Missing^a^* | *8 (6)* | *0 (0)* |  |
| **Age** | Mean (SD) | 36.6 (9.6) | 37.0 (7.9) | 0.01 |
| **Facility type/level** | HC II | 28 (18) | 25 (28) | <0.01 |
|  | HC III | 79 (60) | 43 (47) |  |
|  | HCIV | 24 (22) | 23 (25) |  |
| **Cadre** | Clinicians MO or CO | 23 (18) | 25 (27) | 0.29 |
|  | Nurse or Midwife | 66 (50) | 40 (44) |  |
|  | Nursing assistant /aide | 34 (26) | 23 (26) |  |
|  | Other | 8 (6) | 3 (3) |  |
| **Experience with patients: In last three months, how many patients have you seen with:** | | | | |
| **Hypertension** | *None* | 3 (2) | 0 (0) | 0.23 |
|  | *1-5 patients* | 32 (25) | 18 (20) |  |
|  | *>5 patients* | 96 (73) | 73 (80) |  |
| **Diabetes** | *None* | 28 (21) | 11 (12) | 0.20 |
|  | *1-5 patients* | 59 (38) | 37 (41) |  |
|  | *>5 patients* | 54 (41) | 43 (47) |  |
| **Chronic heart failure** | *None* | 107 (82) | 79 (87) | 0.48 |
|  | *1-5 patients* | 20 (15) | 11 (12) |  |
|  | *>5 patients* | 4 (3) | 1 (1) |  |
| **HIV** | *None* | 8 (6) | 5 (6) | < 0.01 |
|  | *1-5 patients* | 47 (36) | 14 (15) |  |
|  | *>5 patients* | 76 (58) | 72 (79) |  |
| **COPD** | *None* | 102 (78) | 68 (75) | 0.30 |
|  | *1-5 patients* | 24 (18) | 22 (24) |  |
|  | *>5 patients* | 5 (4) | 1 (1) |  |
| **Asthma** | *None* | 35 (27) | 20 (22) | 0.50 |
|  | *1-5 patients* | 90 (69) | 64 (70) |  |
|  | *>5 patients* | 6 (4) | 7 (8) |  |
| **Epilepsy** | *None* | 40 (31) | 16 (18) | 0.01 |
|  | *1-5 patients* | 71 (54) | 48 (53) |  |
|  | *>5 patients* | 20 (15) | 27 (29) |  |
| **Level of comfort: How comfortable are you with patients with** | | | | |
| **Hypertension** | *Very comfortable* | 40 (31) | 28 (31) | 0.96 |
|  | *OK, but need more training* | 89 (68) | 62 (68) |  |
|  | *Very uncomfortable* | 2 (2) | 1 (1) |  |
| **Diabetes** | *Very comfortable* | 33 (25) | 18 (20) | 0.37 |
|  | *OK, but need more training* | 92 (70) | 71 (78) |  |
|  | *Very uncomfortable* | 6 (5) | 2 (2) |  |
| **Chronic heart failure** | *Very comfortable* | 2 (2) | 2 (2) | 0.34 |
|  | *OK, but need more training* | 84 (64) | 66 (73) |  |
|  | *Very uncomfortable* | 45 (34) | 23 (25) |  |
| **HIV** | *Very comfortable* | 50 (38) | 30 (33) | 0.65 |
|  | *OK, but need more training* | 77 (59) | 59 (65) |  |
|  | *Very uncomfortable* | 4 (3) | 2 (2) |  |
| **COPD** | *Very comfortable* | 7 (5) | 2 (2) | 0.21 |
|  | *OK, but need more training* | 80 (61) | 65 (72) |  |
|  | *Very uncomfortable* | 44 (34) | 24 (26) |  |
| **Asthma** | *Very comfortable* | 42 (32) | 24 (26) | 0.20 |
|  | *OK, but need more training* | 81 (62) | 65 (72) |  |
|  | *Very uncomfortable* | 8 (6) | 2 (2) |  |
| **Epilepsy** | *Very comfortable* | 18 (14) | 16 (17) | 0.64 |
|  | *OK, but need more training* | 98 (75) | 67 (74) |  |
|  | *Very uncomfortable* | 15 (11) | 8 (9) |  |
| **Necessary equipment: Do you have the necessary equipment to manage** | | | | |
| **Hypertension** | *Yes, I have all* | 103 (79) | 53 (58) | < 0.01 |
|  | *No, I lack some equipment* | 25 (19) | 38 (42) |  |
|  | *I don’t know* | 3 (2) | 0 (0) |  |
| **Diabetes** | *Yes, I have all* | 85 (65) | 21 (23) | <0.01 |
|  | *No, I lack some equipment* | 42 (32) | 70 (77) |  |
|  | *I don’t know* | 4 (3) | 0 (0) |  |
| **HIV** | *Yes, I have all* | 79 (60) | 53 (58) | 0.55 |
|  | *No, I lack some equipment* | 48 (37) | 37 (41) |  |
|  | *I don’t know* | 4 (3) | 1 (1) |  |
| **Necessary drugs: Do you have the necessary drugs to manage** | | | | |
| **Hypertension** | *Yes, I have all* | 96 (73) | 19 (21) | < 0.01 |
|  | *No, I lack the drugs* | 31 (24) | 72 (79) |  |
|  | *I don’t know* | 4 (3) | 0 (0) |  |
| **Diabetes** | *Yes, I have all* | 76 (58) | 16 (18) | <0.01 |
|  | *No, I lack the drugs* | 51 (39) | 75 (82) |  |
|  | *I don’t know* | 4 (3) | 0 (0) |  |
| **HIV** | *Yes, I have all* | 99 (75) | 55 (60) | 0.04 |
|  | *No, I lack the drugs* | 30 (23) | 35 (39) |  |
|  | *I don’t know* | 2 (2) | 1 (1) |  |
| Support supervision and training: When last did you have detailed advice or training through | | | | |
| **For hypertension or diabetes:** |  |  |  |  |
| **a facility supervisor** | *Within last year* | 69 (53) | 30 (33) | < 0.01 |
|  | *Before last year* | 22 (17) | 35 (39) |  |
|  | *Never* | 29 (22) | 15 (16) |  |
|  | *I am in charge at this facility* | 11 (8) | 11 (12) |  |
| **district health management or other department** | *Within last year* | 58 (44) | 18 (20) | < 0.01 |
|  | *Before last year* | 25 (19) | 29 (32) |  |
|  | *Never* | 48 (37) | 44 (48) |  |
| **a training course** | *Within last year* | 51 (39) | 21 (23) | < 0.01 |
|  | *before last year* | 34 (26) | 41 (45) |  |
|  | *Never* | 46 (35) | 29 (32) |  |
| **For HIV:** |  |  |  |  |
| **a facility supervisor** | *Within last year* | 62 (48) | 45 (49) | 0.92 |
|  | *Before last year* | 33 (25) | 20 (22) |  |
|  | *Never* | 24 (18) | 16 (18) |  |
|  | *I am in charge at this facility* | 12 (9) | 10 (11) |  |
| **district health management or other department** | *Within last year* | 72 (55) | 37 (41) | 0.04 |
|  | *Before last year* | 28 (21) | 33 (36) |  |
|  | *Never* | 31 (24) | 21 (23) |  |
| **a training course** | *Within last year* | 40 (31) | 27 (30) | 0.88 |
|  | *before last year* | 59 (45) | 39 (43) |  |
|  | *Never* | 32 (24) | 25 (27) |  |

^1^Pvalue from Pearson chi-squared statistic with the second-order correction of Rao and Scott to account for the clustered design

^a^Missing record on gender for 8 HWs in 2016
