# Supplementary material for: Medium-to-long term sustainability of a health systems intervention to improve service readiness and quality of non-communicable disease (NCD) patient care and experience at primary care settings in Uganda
Source: BMC Health Serv Res. 2023 Sep 22;23:1022. doi: 10.1186/s12913-023-09983-7 (PMC10514956; doi:10.1186/s12913-023-09983-7)
Supplement: Supplementary file 2 — Additional file 2: Supplementary table 2.- Facility inspection - distribution of the constituents of the elements of service availability and readiness (SAR). [file 12913_2023_9983_MOESM2_ESM.docx]

Supplementary table 2 - Facility inspection - distribution of the constituents of the elements of service availability and readiness (SAR)

| **Element of service availability and readiness (SAR)** | | | **2016 n (%) (N=22)** | | | **2020 n (%) (N=22)** | | | **p-value^1^** | |  |
| --- | --- | --- | --- | --- | --- | --- | --- | --- | --- | --- | --- |
| 1. **Availability of basic equipment** | |  | | |  | | |  | | | |
| **BP machine** | *missing from OPD* | | 0 (0) | | | 1 (5) | | | 1.00 | |  |
|  | *1 available not working* | | 1 (5) | | | 0 (0) | | |  |  |  |
|  | *1 available and working* | | 12 (55) | | | 11 (50) | | |  |  |  |
|  | *2 or more machines available and working* | | 9 (40) | | | 10 (45) | | |  |  |  |
| **BP Cuffs** | *cuffs missing* | | 0 (0) | | | 1 (5) | | | 0.20 | |  |
|  | *standard size only* | | 5 (23) | | | 9 (40) | | |  |  |  |
|  | *At least 2 cuff sizes* | | 17 (77) | | | 12 (55) | | |  |  |  |
| **Adult weight scale** | *available not working* | | 0 (0) | | | 2 (9) | | | **<0.01** | |  |
|  | *available and working* | | 0 (0) | | | 8 (36) | | |  |  |  |
|  | *evidence of use from records* | | 22 (100) | | | 12 (55) | | |  |  |  |
| **Adult size stethoscope** | *missing from OPD* | | 10 (45) | | | 11 (50) | | | 0.61 | |  |
|  | *1 available not working* | | 4 (18) | | | 1 (5) | | |  |  |  |
|  | *1 available and working* | | 7 (32) | | | 8 (36) | | |  |  |  |
|  | *2 or more machines available and working* | | 1 (5) | | | 2 (9) | | |  |  |  |
| **Stadiometer** | *missing from OPD* | | 0 (0) | | | 1 (5) | | | **<0.01** | |  |
|  | *available and working* | | 1 (5) | | | 17 (77) | | |  |  |  |
|  | *evidence of use from records* | | 21 (95) | | | 4 (18) | | |  |  |  |
| **Glucometers for HCIVs & HCIIIs**^1^ | *missing from OPD* | | 0 (0) | | | 1 (8) | | | 0.10 | |  |
|  | *available and working* | | 4 (31) | | | 0 (0) | | |  |  |  |
|  | *evidence of use from records* | | 9 (69) | | | 12 (92) | | |  |  |  |
| **Blood glucose strips for HCIVs/HCIIIs^2^** | *missing from OPD* | | 0 (0) | | | 1 (8) | | | **0.02** | |  |
|  | *some strips available* | | 0 (0) | | | 5 (38) | | |  |  |  |
|  | *supply for at least one month* | | 13 (100) | | | 7 (54) | | |  |  |  |
| **Urine glucose strips for HCIIs^3^** | *missing from OPD* | | 0 (0) | | | 16 (72) | | | **<0.01** | |  |
|  | *have at least 30 tests* | | 0 (0) | | | 5 (23) | | |  |  |  |
|  | *have more than 30 tests* | | 22 (100) | | | 1 (5) | | |  |  |  |
| **Patient register** | *available but not used* | | 0 (0) | | | 1 (5) | | | 0.22 | |  |
|  | *available and used but not up-to-date* | | 6 (27) | | | 10 (45) | | |  |  |  |
|  | *available and up-to-date* | | 16 73) | | | 11 (50) | | |  |  |  |
| **HT / DM screening logbook** | *missing form OPD* | | 0 (0) | | | 3 (13) | | | **<0.01** | |  |
|  | *available but not used* | | 0 (0) | | | 1 (5) | | |  |  |  |
|  | *available evidence of some use* | | 1 (5) | | | 12 (55) | | |  |  |  |
|  | *available, evidence of good use* | | 21 (95) | | | 6 (27) | | |  |  |  |
| **Referral register** | *missing form OPD* | | 8 (36) | | | 13 (59) | | | 0.27 | |  |
|  | *available but not used* | | 1 (5) | | | 1 (5) | | |  |  |  |
|  | *available, used but incorrectly* | | 3 (14) | | | 4 (18) | | |  |  |  |
|  | *available and used correctly* | | 10 (45) | | | 18 (4) | | |  |  |  |
| 1. **Availability of essential drugs** | | | | | | | | | | | |
| **Essential drugs for HT** |  | | |  | | |  | | |  | |
| HCIVs and HCIIIs^2^ | *1st line drugs available* | | | 4 (31) | | | 8 (61) | | | 0.39 | |
|  | *2nd line drugs also available* | | | 7 (54) | | | 4 (31) | | |  |  |
|  | *3rd line drugs also available* | | | 2 (15) | | | 1 (8) | | |  |  |
| HCIIs^3^ | *missing 1st line drugs* | | | 0 (0) | | | 2 (22) | | | 0.27 | |
|  | *1st line drugs available for <1 month* | | | 1 (13) | | | 3 (33) | | |  |  |
|  | *1st line drugs available for 1-3 month* | | | 6 (74) | | | 3 (33) | | |  |  |
|  | *1st line drugs available for >3months* | | | 1 (13) | | | 1 (12) | | |  |  |
| **Essential drugs for DM** |  | | |  | | |  | | |  | |
| HCIVs and HCIII^2^ | *1st line drugs available* | | | 12 (92) | | | 10 (77) | | | 0.59 | |
|  | *2nd line drugs also available* | | | 1 (8) | | | 3 (23) | | |  |  |
| HCIIs^3^ | *missing 1st line drugs* | | | 0 (0) | | | 5 (56) | | | **0.03** | |
|  | *1st line drugs available* | | | 9 (100) | | | 4 (55) | | |  |  |
| 1. **Quality of records** | | | | | | | | | | | |
| **NCD register**^4^ | *names recorded* | | | 22 (100) | | | 22 (100) | | | 1.00 | |
|  | *age and sex recorded* | | | 22 (100) | | | 22 (100) | | | 1.00 | |
|  | *residence recorded* | | | 16 (73) | | | 21 (95) | | | 0.10 | |
|  | *initial treatment recorded* | | | 22 (100) | | | 21 (95) | | | 1.00 | |
| **NCD follow-up record**^4^ | *age and sex recorded* | | | 19 (86) | | | 22 (100) | | | 1.00 | |
|  | *bp/blood glucose level recorded at last visit* | | | 22 (100) | | | 22 (100) | | | 1.00 | |
|  | *risk factors recorded* | | | 14 (64) | | | 19 (86) | | | 0.16 | |
| **Referral register book** | *missing from OPD* | | | 8 (36) | | | 13 (59) | | | 0.24 | |
|  | *available not used* | | | 1 (5) | | | 1 (5) | | |  |  |
|  | *available and used but incorrectly* | | | 3 (14) | | | 4 (18) | | |  |  |
|  | *available and used correctly* | | | 10 (45) | | | 4 (18) | | |  |  |
| **Health education record book** | *missing from OPD* | | | 1 (5) | | | 10 (45) | | | 0.20 | |
|  | *available no evidence* | | | 0 (0) | | | 5 (23) | | |  |  |
|  | *available, evidence of some use* | | | 12 (55) | | | 5 (23) | | |  |  |
|  | *evidence of regular use* | | | 2 (9) | | | 2 (9) | | |  |  |
|  | *ncd-related health education given* | | | 7 (31) | | | 0 (0) | | |  |  |
| 1. **Utilisation of the health facility** | | | | | | | | | | | |
| ***Number of newly registered patients over last 3 months*** | | | |  | | |  | | |  | |
| with HT at HCIIIs & HCIVs^2^ | *1-20 patients* | | | 0 (0) | | | 9 (69) | | | **<0.01** | |
|  | *21-40 patients* | | | 3 (23) | | | 1 (8) | | |  |  |
|  | *>40 patients* | | | 10 (77) | | | 3 (3) | | |  |  |
| with HT at HCIIs^3^ | *1-10 patients* | | | 0 (0) | | | 8 (89) | | | **<0.01** | |
|  | *11-20 patients* | | | 0 (0) | | | 1 (11) | | |  |  |
|  | *>20 patients* | | | 9 (100) | | | 0 (0) | | |  |  |
| with DM at HCIIIs & HCIVs^2^ | *no patients registered* | | | 0 (0) | | | 0 (0) | | | **<0.01** | |
|  | *1- 4 patients* | | | 2 (15) | | | 9 (70) | | |  |  |
|  | *5-10 patients* | | | 1 (8) | | | 2 (15) | | |  |  |
|  | *>10 patients* | | | 10 (77) | | | 2 (15) | | |  |  |
| with DM at HCIIs^3^ | *no patients registered* | | | 1 (11) | | | 7 (78) | | | **0.01** | |
|  | *1- 2 patients* | | | 2 (22) | | | 2 (22) | | |  |  |
|  | *3-5 patients* | | | 4 (45) | | | 0 (0) | | |  |  |
|  | *>5 patients* | | | 2 (22) | | | 0 (0) | | |  |  |
| ***Evidence of utilisation over last one year*** | | | |  | | |  | | |  | |
| for HCIIIs & HCIVs^2^ | *none or <10% increase* | | | 2 (15) | | | 8 (61) | | | **0.05** | |
|  | *increase of 10-100%* | | | 8 (62) | | | 4 (31) | | |  |  |
|  | *increase of >100%* | | | 3 (23) | | | 1 (8) | | |  |  |
| for HCIIs^3^ | *none or <10% increase* | | | 2 (22) | | | 7 (78) | | | **<0.05** | |
|  | *increase of 10-100%* | | | 3 (33) | | | 2 (22) | | |  |  |
|  | *increase of >100%* | | | 4 (45) | | | 0 (0) | | |  |  |
| 1. **Preventive activities** | | | | | | | | | | | |
| **Health education session provided in waiting area** | *no evidence* | | | 1 (5) | | | 4 (18) | | | 0.25 | |
|  | *at least one session verbally reported* | | | 11 (50) | | | 6 27) | | |  |  |
|  | *at least one session recorded* | | | 2 (9) | | | 6 27) | | |  |  |
|  | *1-4 per month* | | | 6 (27) | | | 4 (18) | | |  |  |
|  | *more than 1 per week* | | | 2 (9) | | | 2 (9) | | |  |  |
| **Screening session for HT or DM conducted** | *no evidence* | | | 0 (0) | | | 3 (14) | | | **<0.01** | |
|  | *at least one screening session verbally reported* | | | 0 (0) | | | 3 (14) | | |  |  |
|  | *at least one screening session recorded* | | | 0 (0) | | | 9 (41) | | |  |  |
|  | *10-50 patients screened* | | | 8 (36) | | | 7 (32) | | |  |  |
|  | *>50 patients screened* | | | 14 (64) | | | 0 (0) | | |  |  |
| **Outreach activities** | *None* | | | 3 (14) | | | 20 (91) | | | **<0.01** | |
|  | *1-2 per month* | | | 1 (5) | | | 1 (5) | | |  |  |
|  | *more than 2 per month* | | | 18 (81) | | | 1 (5) | | |  |  |

**Footnote**

^1^P-value from Fisher’s statistic with the second-order correction of Rao and Scott to account for the clustered design

^2^Only applied to HCIIIs and HCIVs

^3^Only applied to HCIIs

^4^Applied to all health facilities
